# DDO1002, an NRF2–KEAP1 inhibitor, improves hematopoietic stem cell aging and stress response

**DOI:** 10.1093/lifemedi/lnae043

**Published:** 2024-12-12

**Authors:** Yuwen Li, Aiwei Wu, Xinrong Jin, Haiping Shen, Chenyan Zhao, Xiao Yi, Hui Nie, Mingwei Wang, Shouchun Yin, Hongna Zuo, Zhenyu Ju, Zhenyu Jiang, Hu Wang

**Affiliations:** Zhejiang Key Laboratory of Medical Epigenetics, School of Basic Medical Sciences, The Third People’s Hospital of Deqing, Department of Cardiology, Affiliated Hospital of Hangzhou Normal University, Hangzhou Normal University, Hangzhou 311121, China; Zhejiang Key Laboratory of Medical Epigenetics, School of Basic Medical Sciences, The Third People’s Hospital of Deqing, Department of Cardiology, Affiliated Hospital of Hangzhou Normal University, Hangzhou Normal University, Hangzhou 311121, China; Zhejiang Key Laboratory of Medical Epigenetics, School of Basic Medical Sciences, The Third People’s Hospital of Deqing, Department of Cardiology, Affiliated Hospital of Hangzhou Normal University, Hangzhou Normal University, Hangzhou 311121, China; Zhejiang Key Laboratory of Medical Epigenetics, School of Basic Medical Sciences, The Third People’s Hospital of Deqing, Department of Cardiology, Affiliated Hospital of Hangzhou Normal University, Hangzhou Normal University, Hangzhou 311121, China; Zhejiang Key Laboratory of Medical Epigenetics, School of Basic Medical Sciences, The Third People’s Hospital of Deqing, Department of Cardiology, Affiliated Hospital of Hangzhou Normal University, Hangzhou Normal University, Hangzhou 311121, China; Zhejiang Key Laboratory of Medical Epigenetics, School of Basic Medical Sciences, The Third People’s Hospital of Deqing, Department of Cardiology, Affiliated Hospital of Hangzhou Normal University, Hangzhou Normal University, Hangzhou 311121, China; Zhejiang Key Laboratory of Medical Epigenetics, School of Basic Medical Sciences, The Third People’s Hospital of Deqing, Department of Cardiology, Affiliated Hospital of Hangzhou Normal University, Hangzhou Normal University, Hangzhou 311121, China; Zhejiang Key Laboratory of Medical Epigenetics, School of Basic Medical Sciences, The Third People’s Hospital of Deqing, Department of Cardiology, Affiliated Hospital of Hangzhou Normal University, Hangzhou Normal University, Hangzhou 311121, China; Zhejiang Key Laboratory of Medical Epigenetics, School of Basic Medical Sciences, The Third People’s Hospital of Deqing, Department of Cardiology, Affiliated Hospital of Hangzhou Normal University, Hangzhou Normal University, Hangzhou 311121, China; Zhejiang Key Laboratory of Medical Epigenetics, School of Basic Medical Sciences, The Third People’s Hospital of Deqing, Department of Cardiology, Affiliated Hospital of Hangzhou Normal University, Hangzhou Normal University, Hangzhou 311121, China; MOE Key Laboratory of Regenerative Medicine, Institute of Aging and Regenerative Medicine, Jinan University, Guangzhou 510632, China; Jiang Su Key Laboratory of Drug Design and Optimization and State Key Laboratory of Natural Medicines, Department of Medicinal Chemistry, School of Pharmacy, China Pharmaceutical University, Nanjing 210009, China; Zhejiang Key Laboratory of Medical Epigenetics, School of Basic Medical Sciences, The Third People’s Hospital of Deqing, Department of Cardiology, Affiliated Hospital of Hangzhou Normal University, Hangzhou Normal University, Hangzhou 311121, China

**Keywords:** hematopoietic stem cell, NRF2, aging, reactive oxygen species

## Abstract

Oxidative stress diminishes the functionality of hematopoietic stem cells (HSCs) as age advances, with heightened reactive oxygen species (ROS) levels exacerbating DNA damage, cellular senescence, and hematopoietic impairment. DDO1002, a potent inhibitor of the NRF2–KEAP1 pathway, modulates the expression of antioxidant genes. Yet, the extent to which it mitigates hematopoietic decline post-total body irradiation (TBI) or in the context of aging remains to be elucidated. Our study has elucidated the role of DDO1002 in modulating NRF2 activity, which, in turn, activates the NRF2-driven antioxidant response element (ARE) signaling cascade. This activation can diminish intracellular levels of ROS, thereby attenuating cellular senescence. In addition, DDO1002 has been demonstrated to ameliorate DNA damage and avert HSC apoptosis, underscoring its potential to mitigate hematopoietic injury precipitated by TBI. Competitive transplantation assay revealed that the administration of DDO1002 can improve the reconstitution and self-renewal capacity of HSCs in aged mice. Single-cell sequencing analysis elucidated that DDO1002 treatment attenuated intracellular inflammatory signaling pathways and mitigated ROS pathway in aged HSCs, suggesting its potential to restore the viability of these cells. Consequently, DDO1002 effectively activated the NRF2–ARE pathway, delaying cellular senescence and ameliorating impaired hematopoiesis, thereby demonstrating its potential as a therapeutic agent for age-related hematopoietic disorders.

## Introduction

Hematopoietic stem cells (HSCs) possess the remarkable capability for self-renewal and multilineage differentiation, giving rise to a spectrum of mature blood and immune cells essential for physiological functions [1, 2]. The process of HSC differentiation is bifurcated: the initial phase involves primary differentiation into bone marrow (BM) cells, while the subsequent phase encompasses terminal differentiation into fully mature blood cells within the bloodstream or peripheral tissues. Oxidative stress, a critical cellular stressor, is characterized by an elevation in reactive oxygen species (ROS) levels and the consequent accumulation of oxidative stress byproducts. This surge in ROS and oxidative damage can precipitate a cascade of detrimental cellular responses, including DNA damage, cell cycle dysregulation, premature cell senescence, and, ultimately, the impairment of HSC function [1, 2]. These effects underscore the pivotal role of oxidative stress in the etiology of various hematopoietic disorders and the potential therapeutic targets for mitigating its impact on HSC health.

The transcription factor NRF2 is composed of 605 amino acids and contains seven highly conserved regions, Neh1–Neh7, each of which has a unique function in controlling diverse transcriptional activities [3]. The Neh2 domain specifically interacts with the Kelch domain of Kelch-like ECH-associated protein 1 (KEAP1) to mediate the ubiquitination and degradation of NRF2 [3–5]. KEAP1 is composed of three functional domains and is responsible for protein ubiquitination. NRF2 regulates oxidative stress by regulating the expression of more than 250 genes [6]. The antioxidant response element (ARE), which is located in the promoter region of downstream target genes, is regulated by NRF2. NRF2 binds to the sMaf protein to form a heterodimer that recognizes and binds to the ARE sequence, thereby activating downstream gene expression, particularly that of various antioxidant proteins, metabolism-related enzymes, and inflammatory factors [7]. The disruption of NRF2-mediated antioxidative response is the driving force for premature aging [8]. Therefore, the development and design of drugs targeting NRF2–KEAP1 interaction has potential for the effective treatment of various age-related disorders.

Traditional NRF2 activators, or more precisely, KEAP1 inhibitors, can change the conformation of KEAP1 by covalently modifying the cysteine residues on KEAP1, thereby allowing NRF2 to escape ubiquitination degradation [9–11]. Most NRF2 activators are plant-derived natural compounds derived from various plants. Dimethyl fumarate is an effective NRF2 activator that can promote NRF2 activation by reacting with Cys151 on KEAP1 to inhibit NRF2 ubiquitination and degradation [10], thereby playing a role in alleviating inflammation, oxidative stress, and ferroptosis [12]. Sulforaphane is a natural NRF2 activator that can react with KEAP1 Cys151 and exhibits a therapeutic effect on type II diabetes and its complications [13]. Several electrophilic synthetic organic compounds have been developed to mimic NRF2 activation. These activators inhibit NRF2 ubiquitination by reacting with Cys residues in KEAP1, thereby inducing NRF2 activation. For example, bardoxolone methyl (CDDO-Me) [14] has potential therapeutic effects on inflammation-related diseases, as well as remission effects on chronic kidney disease and type II diabetes [15].

Inhibitors of NRF2–KEAP1 protein–protein interaction (PPI) represent a new type of NRF2 activator that blocks the interaction between KEAP1 and NRF2, thereby allowing NRF2 to escape ubiquitination and degradation. KEAP1 binds structurally to the ETGE and DLG motifs of NRF2 via its Kelch domain, and NRF2 interacts with the KEAP1 cavity, which is divided into six sub-pockets (P1–P6) [16]. The binding of KEAP1–Kelch to NRF2–ETGE relies on the interaction between the P1 and P2 sub-pockets and two key glutamic acid residues, Glu79 and Glu82 [17]. This binding mode of KEAP1 and NRF2 inspired the design of NRF2–KEAP1 PPI inhibitors that compete for the binding site of NRF2 on KEAP1 by simulating the interaction between NRF2 and KEAP1 subpockets, particularly P1 and P2. This novel NRF2 activator is safer and more selective than conventional electrophilic activators and may modify redox-sensitive cysteine residues in other functional proteins. Efficient small-molecule inhibitors of NRF2–KEAP1 PPI can alleviate oxidative stress and disease progression by activating NRF2. For example, NXPZ-2 can directly inhibit Nrf2–Keap1 protein interactions, upregulate the expression of antioxidant genes downstream of Nrf2, and exert neuroprotective activity [18]. CPUY192018 activates the expression of its downstream antioxidant genes and reduces the expression of inflammatory factors such as TNF-α, IFN-γ, IL-6, and IL-1β by upregulating Nrf2 to alleviate ulcerative colitis and inflammatory kidney disease [19]. Our previously designed potent NRF2–KEAP1 PPI inhibitor, DDO1002, could activate the expression of NRF2-mediated cytoprotective genes at the mRNA level [17]. NRF2 is widely expressed in various tissues, but its transcriptional characteristics vary in different tissues and cells; thus, NRF2 activators may exhibit different pharmacokinetic characteristics in different tissues.

NRF2 also plays a role in the hematopoietic system. For example, the HSCs of Nrf2-knockout mice are more sensitive to oxidative stress, and HSC numbers are increased by sacrificing the resting state and self-renewal of HSCs, eventually leading to HSC exhaustion [20, 21]. In addition, NRF2 protects mesenchymal stem cells (MSCs), megakaryocytes, T cells, and other cells in the niche from oxidative stress, maintaining a suitable niche microenvironment for HSCs [22]. Thus, small-molecule antioxidants targeting NRF2 may be a viable approach for mitigating aging-related hematopoietic dysfunction. Vam3 is a small-molecule compound extracted from golden grapes that can improve the self-renewal and differentiation of hematopoietic progenitor cells and HSCs by regulating Nrf2, leading to the alleviation of hematopoietic system dysfunction in irradiated mice [23]. However, whether KEAP1–NRF2 PPI inhibitors exert a protective effect on the hematopoietic system aging remains unclear.

Therefore, in this study, we determined the role of the NRF2–KEAP1 PPI inhibitor DDO1002 in the hematopoietic system aging. Specifically, we showed that DDO1002 effectively activates the expression of Nrf2 and Nrf2-mediated antioxidative responses in both cellular and mouse models. Moreover, DDO1002 significantly decreased the ROS levels and alleviated cell senescence. We conducted an investigation into the therapeutic potential of DDO1002 on the recovery from hematopoietic damage associated with ionizing radiation and the aging process. Our findings demonstrate that DDO1002 significantly ameliorates hematopoietic impairment through the activation of the Nrf2–ARE signaling pathway. This activation is instrumental in counteracting the deleterious effects of oxidative stress and promoting the restoration of hematopoietic function. The study underscores the significance of DDO1002 as a promising candidate for drug development, targeting the treatment of hematopoietic dysfunction induced by irradiation, and age-related hematopoietic disorders. The modulation of the NRF2–ARE pathway by DDO1002 offers a novel therapeutic strategy that could potentially mitigate the adverse effects of radiation exposure and the decline in hematopoietic capacity observed with aging.

## Results

### DDO1002 upregulates NRF2 in senescent cells and activates the ARE pathway

**Figure 1.**
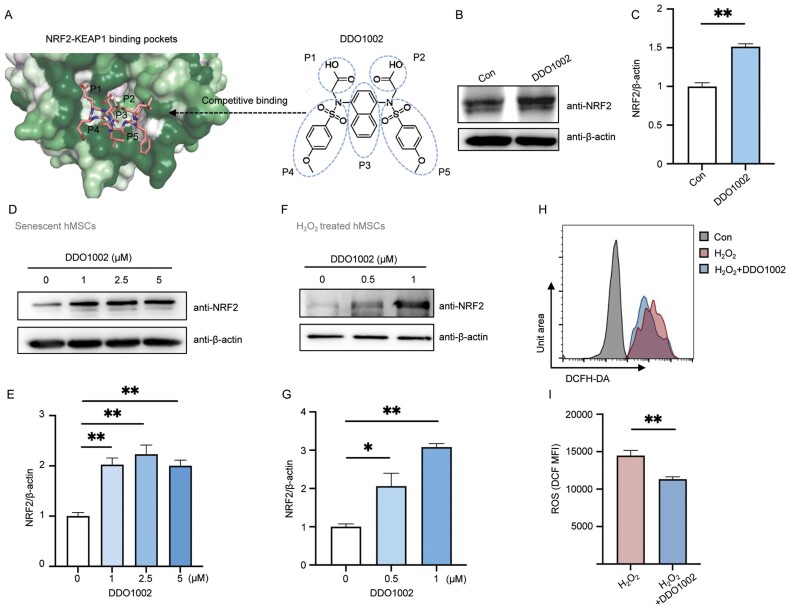
**DDO1002 upregulates NRF2 in senescent cells.** (A) DDO1002 was designed to competitively bind to the surface of KEAP1−NRF2 PPI. NRF2 peptides are displayed as sticks, and the surface of KEAP1 is partially charged. (B, C) Relative protein levels of NRF2 in Control (Con) and DDO1002-treated (DDO1002) hMSCs. (D, E) Relative protein levels of NRF2 in Control (Con) and DDO1002-treated (DDO1002) replicative senescent hMSCs. (F, G) Relative protein levels of NRF2 in Control (Con) and DDO1002-treated (DDO1002) H_2_O_2_-induced senescent cells. (H, I) The reactive oxygen species (ROS) levels of hMSCs (control, H_2_O_2_-treated, and H_2_O_2_ + DDO1002-treated) were assessed using 2,7-dichlorodihydrofluorescein (DCF) by flow cytometry. The bar graph represents the mean fluorescence intensity (MFI), and the values are presented as the mean ± SEM of MFI (*n* = 3).

According to the molecular determinants of KEAP1 binding, a structure-based NRF2−KEAP1 PPI inhibitor, DDO1002, was designed to occupy five sub-pockets (P1–P5) of KEAP1 (Fig. 1A). This leads to termination of the NRF2–KEAP1 interaction. To verify whether DDO1002 could effectively activate NRF2, we tested the effect of DDO1002 on human umbilical cord MSCs (hMSCs). The results showed that DDO1002 significantly upregulated NRF2, indicating that DDO1002 can effectively inhibit KEAP1-mediated NRF2 ubiquitination degradation and promote significant NRF2 accumulation in cells (Fig. 1B and 1C). A concentration gradient of 1–5 μM was used to treat hMSCs that had passed to senescence, which showed that the protein expression levels of NRF2 could be activated at low concentrations (Fig. 1D and 1E). Considering that oxidative stress caused by intracellular stimuli can induce cell senescence, we treated hydrogen peroxide (H_2_O_2_)-induced senescent hMSCs with DDO1002 to verify whether NRF2 could be activated. DDO1002 significantly increased NRF2 expression in H_2_O_2_-induced senescent cells and activated the protein expression of NRF2 at low concentrations (Fig.1F and 1G). As an important antioxidant factor, NRF2 activation reduces ROS levels and prevents oxidative stress. To investigate whether DDO1002 can alleviate intracellular oxidative stress, we measured ROS levels in hMSCs after H_2_O_2_ and subsequent DDO1002 treatment to evaluate the effect of DDO1002 on oxidative stress. The results showed that ROS levels significantly increased after H_2_O_2_ treatment, whereas the subsequent use of DDO1002 reduced the abnormal increase in ROS levels in cells, suggesting that DDO1002 could effectively alleviate oxidative stress (Fig. 1H and 1I).

**Figure 2.**
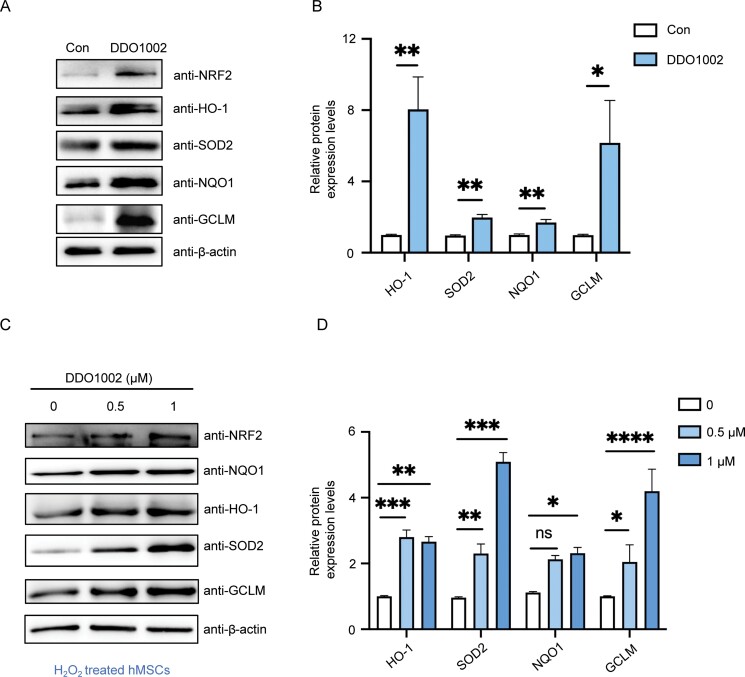
**DDO1002 activates the ARE pathway in senescent cells.** (A) Western blot analysis of NRF2, HO-1, SOD2, NQO-1, and GCLM expression in replicative senescent hMSC*s*. (B) Quantitative analysis of HO-1, SOD2, NQO-1, and GCLM protein levels (*P < 0.05, **P < 0.01, ***P < 0.001 vs. control). (C) Western blot analysis of NRF2, HO-1, SOD2, NQO-1, and GCLM in H_2_O_2_-induced senescent hMSCs. (D) Quantitative analysis of HO-1, SOD2, NQO-1, and GCLM protein levels (*P < 0.05, **P < 0.01, ***P < 0.001 vs. control). Values represent the mean ± SEM of triplicate independent experiments.

To further test whether DDO1002 can regulate the expression of antioxidant genes after activation of NRF2, mRNA and protein expression levels of the downstream genes heme oxygenase-1 (*HO-1*), NAD(P)H dehydrogenase quinone 1 (*NQO1*), glutamate-cysteine ligase modifier subunit (*GCLM*), and superoxide dismutase 2 (*SOD2*) were detected in senescent cells after treatment with DDO1002. Gene expression analysis using fluorescent real-time quantitative PCR and western blotting confirmed the upregulated expression of these antioxidant genes (Fig. 2A , 2B, S1A–C). Similarly, NRF2-mediated antioxidant gene expression gradually increased after treatment with 0.5 μM or 1 μM of DDO1002 in H_2_O_2_-induced senescent cells (Fig. 2C, 2D and S1D). However, the expression of these antioxidant genes showed no significant changes when NRF2 was knocked down in DDO1002-treated cells (Fig. S1E). These results indicate that DDO1002 regulates the expression of downstream antioxidant genes by upregulating the expression of NRF2.

### DDO1002 delays cellular senescence

**Figure 3.**
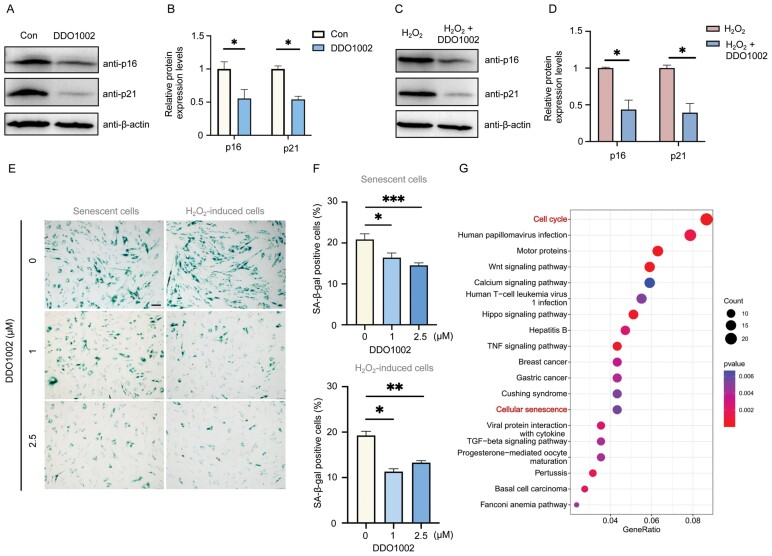
**DDO1002 delays cell senescence.** (A) Relative protein levels of p21 and p16 in control and DDO1002-treated senescent hMSCs. (B) Quantitative analysis of p21 and p16 protein levels (*P < 0.05, **P < 0.01, vs. control). (C) In H_2_O_2_-induced senescent hMSCs, relative protein levels of p21 and p16 after treatment with DDO1002. (D) In H_2_O_2_-induced senescent hMSCs, quantitative analysis of p21and p16 protein levels after treatment with DDO1002. (*P < 0.05 vs. control). (E) SA-β-gal staining of control and DDO1002-treated cells with a concentration of 1 and 2.5 µM in senescent hMSCs and H_2_O_2_-induced senescent hMSCs. Scale bars: 100 µM. (F) Quantitative analysis of SA-β-gal-positive cells in senescent hMSCs and H_2_O_2_-induced senescent hMSCs. (G) KEGG pathway analysis of DDO1002-treated senescent hMSCs compared to control. The pathways of the cell cycle and cell senescence are highlighted.

NRF2 activation by DDO1002 may upregulate NRF2-mediated antioxidant gene expression. Therefore, we investigated whether DDO1002 could effectively delay cellular senescence. Following DDO1002 treatment of senescent hMSCs, the expression of the senescence-associated proteins p16 and p21 was significantly downregulated (Fig. 3A and 3B). Subsequently, the degree of cellular senescence was determined by β-galactosidase staining of senescent hMSCs. Both the generation of β-galactosidase and the percentage of SA-β-gal-positive cells were significantly reduced (Fig. 3E and 3F). Similarly, DDO1002 also delayed the expression of senescence-related proteins p16 and p21 in H_2_O_2_-treated cells, and SA-β-gal-positive cells were significantly reduced in H_2_O_2_-induced senescent cells after the treatment of DDO1002 (Fig. 3C–F). These results indicate that DDO1002 significantly alleviated cellular senescence.

To further explore the underlying mechanism by which DDO1002 delayed cellular senescence, control, and DDO1002-treated hMSCs were subjected to RNA sequencing (Fig. 3G). KEGG pathway analysis showed that almost 2.5% of differentially expressed genes were enriched in cell senescence and cell cycle pathways, which was consistent with the decrease in SA-β-gal-positive cells after DDO1002 treatment, indicating that treatment with DDO1002 regulated cell cycle and senescence (Fig. 3G). In addition, differential expression analysis identified most antioxidant- and aging-related genes, such as intracellular *SOD2* (log_2_fold-change = 1.17, *P* < 0.05), which is an important antioxidant enzyme that plays a crucial role in oxidative stress resistance [24]. p15, which is downregulated (log_2_fold-change = −0.90, *P* < 0.05), is also a key regulator of cell senescence [25]. These results indicate that DDO1002 upregulates antioxidant genes and downregulates cellular senescence markers, thereby delaying cellular senescence.

### DDO1002 increases the number and function of HSCs and reduces DNA damage and ROS levels, alleviating TBI-induced hematopoietic injury

**Figure 4.**
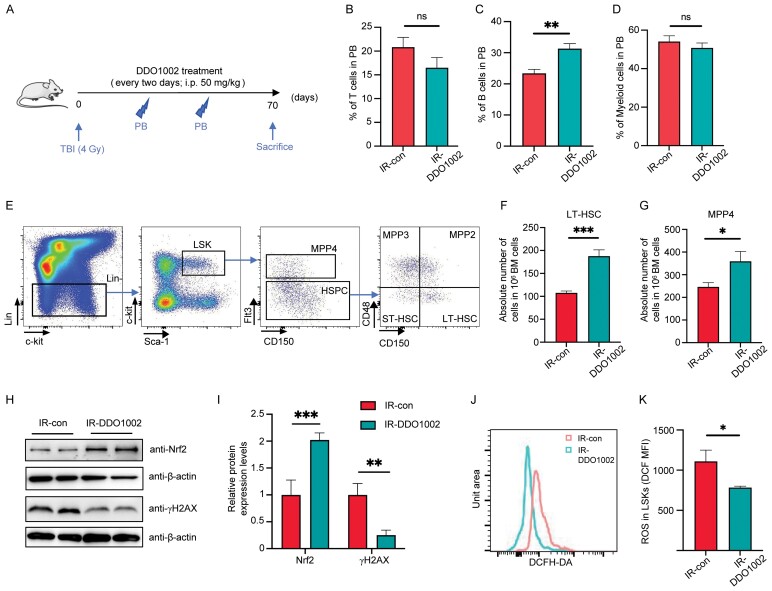
**DDO1002 increases the number and function of HSCs and reduces DNA damage and ROS levels, thus alleviating TBI-induced hematopoietic injury.** (A) Mice were treated with control solution or 50 mg/kg DDO1002 every two days after 4 Gy TBI. Peripheral blood samples were collected regularly for analysis and all mice were sacrificed for bone marrow analysis. (B–D) The percentage of (B) T cells, (C) B cells, and (D) myeloid cells in peripheral blood was analyzed using flow cytometry four weeks after DDO1002 treatment. (E) FACS analysis of HSPCs from IR-control and IR-DDO1002-treated mice. (F, G) Absolute numbers of LT-HSCs and MPP4 in BM cells (per 10^6^ cells) are displayed. (H) Western blot analysis of Nrf2 and γH2AX in BM cells from IR-control and IR-DDO1002-treated mice. (I) Quantitative analysis of Nrf2 and γH2AX protein levels (*P < 0.05, **P < 0.01, ***P < 0.001 vs. control). The values represent mean ± SEM of triplicate independent experiments. (J, K) The ROS levels of BM cells from IR-control and IR-DDO1002-treated mice were analyzed with 2,7-dichlorodihydrofluorescein (DCF) using flow cytometry. The bar graph represents the mean fluorescence intensity (MFI); the values are presented as the mean ± SEM of MFI (*n* = 3).

The hematopoietic system is sensitive to total body irradiation (TBI), which can induce hematopoietic system injury and the accumulation of senescent cells [26, 27]. To evaluate the protective effect of DDO1002 against TBI-induced hematopoietic system injury, a 4-Gy dose of TBI was administered to mice, as previously reported [27], prior to administering DDO10002 or phosphate buffered saline (Fig. 4A). Next, we investigated the effects of DDO1002 on the irradiated mice. The body weights of the mice were continuously monitored and recorded during administration. Although we observed no significant difference in body weight, mice treated with DDO1002 exhibited a decreased spleen index and an increased thymus index (Fig. S2A–C). Thus, DDO1002 may reduce the splenic enlargement induced by TBI and enhance immunity in mice.

To investigate the effect of DDO1002 on hematopoiesis in irradiated mice, we performed peripheral blood analysis. The results showed that the proportion of B cells in the peripheral blood of DDO1002-treated mice significantly increased after 4 weeks of administration, whereas no changes were observed in T cells or myeloid cells (Fig. 4B–D). Regarding the effect of DDO1002 on hematopoietic stem and progenitor cells in the BM cells of irradiated mice, flow cytometry analysis showed that the number of HSCs (Lineage^−^Sca-1^+^c-kit^+^CD150^+^CD48^−^) and MPP4 (Lineage^−^Sca-1^+^c-kit^+^CD135^+^) were significantly increased after DDO1002 treatment (Fig. 4E–G). This suggests that DDO1002 increases the number of HSCs and lymphoid multipotent progenitor cells, alleviates HSC exhaustion in the BM, and promotes HSC recovery.

Ionizing radiation can induce DNA damage, including base damage and DNA strand break [28]. When DNA damage occurs, histone H2AX in the nucleus is phosphorylated to γ-H2AX. Therefore, γ-H2AX protein expression in cells can be used to assess the extent of DNA damage. To verify the effect of DDO1002 on DNA damage, we examined the expression levels of γ-H2AX in BM cells. The expression of γ-H2AX protein in the BM cells of the DDO1002-treated group was significantly lower than that in the irradiated group, suggesting that DDO1002 can significantly reduce the degree of DNA damage in the BM cells of irradiated mice (Fig. 4H and 4I).

The hematopoietic system maintains a hypoxic state to maintain homeostasis. Irradiation-induced oxidative stress is an important cause of hematopoietic stem progenitor cell injury [27, 29]. To evaluate the regulatory effect of DDO1002 on oxidative stress in hematopoietic stem progenitor cells of irradiated mice, flow cytometry was used to detect ROS levels in HSCs. The results showed that ROS levels in Lineage^−^Sca-1^+^c-kit^+^ (LSK) cells of DDO1002-treated mice were significantly reduced, which alleviated oxidative damage to HSCs (Fig. 4J and 4K). We then examined the proportion of HSCs in the irradiated and DDO1002-treated groups. Compared with the irradiation group, apoptosis in LSK cells of DDO1002-treated mice was significantly reduced, which was predominantly reflected by early apoptosis; no significant difference was observed in late apoptosis (Fig. S2D and S2E).

To evaluate whether DDO1002 treatment has any adverse effects, different doses of DDO1002 (25 mg/kg, 50 mg/kg, and 75 mg/kg) were administrated to C57BL/6 mice. The morphology of liver and kidney, as well as indicators related to liver and kidney function were examined after treatment. The results showed that there were no significant differences in the morphology of liver and kidney, serum aspartate aminotransferase (AST), and alanine transaminase (ALT) related to liver function, creatinine (CR) related to kidney function with the increase of dosage (Fig. S3A–E).

### DDO1002 alters hematopoiesis in the spleen and enhances hematopoiesis function in aged mice

**Figure 5.**
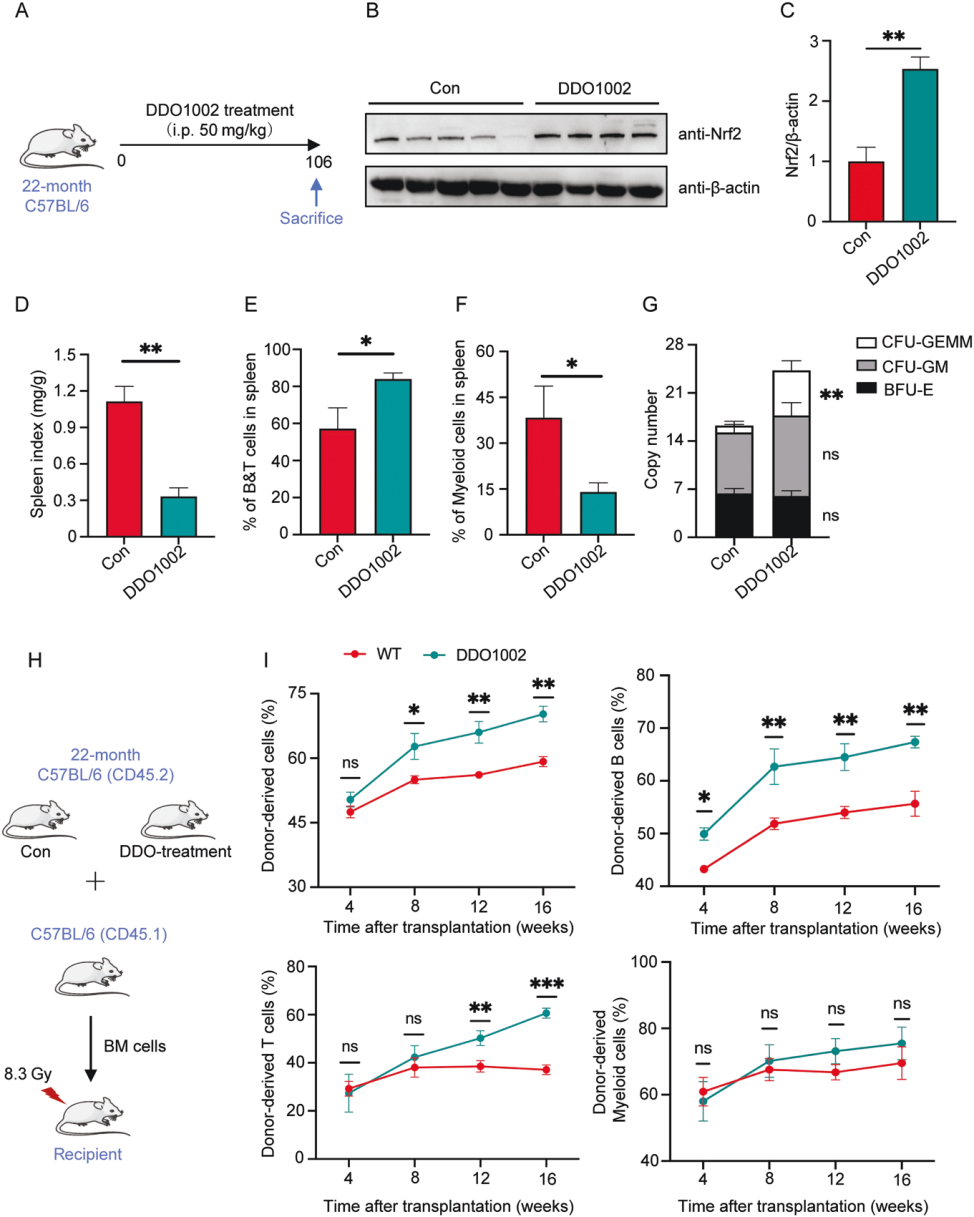
**DDO1002 alters hematopoiesis in the spleen and enhances hematopoiesis function in aged mice.** (A) Aged mice were treated with control solution or 50 mg/kg DDO1002 every two days, and all mice were sacrificed for bone marrow analysis after 106 days. (B) Western blot analysis of Nrf2 in BM cells from control and DDO1002-treated aging mice. (C) Quantitative analysis of Nrf2 protein levels (**P < 0.01 vs. control). The values represent mean ± SEM of triplicate independent experiments. (D) Spleen index (ratio of spleen body weight) of control and DDO1002-treated aging mice. (E, F) The percentages of (E) B and T cells and (F) myeloid cells in the spleen of control and DDO1002-treated aging mice were analyzed by flow cytometry. (G) Colony number of BM cells from control and DDO1002-treated aging mice. Colony forming unit-granulocyte, erythrocyte, monocyte and megakaryocyte (CFU-GEMM), colony forming unit-granulocyte and macrophage (CFU-GM), burst forming unit-erythroid (BFU-E). (H) Schematic for competitive BM transplantation. (I) The percentage of donor-derived PB, B, T, and myeloid cells every four weeks for four months after transplantation.

To explore the effect of DDO1002 on hematopoiesis in aged mice, aged mice were treated with DDO1002 every other day, whereas the control group was treated with phosphate buffered saline (Fig. 5A). After 2 months of continuous administration, the body weight of the aged mice was slightly reduced (Fig. S3F). BM cells were collected to measure Nrf2 protein levels. The results showed that Nrf2 protein levels in the BM cells of DDO1002-treated mice were significantly higher than those in the control group (Fig. 5B and 5C). According to the peripheral blood analysis, DDO1002 significantly increased the number of red blood cells, hemoglobin, and monocytes (Fig. S3G).

In mice, aging is often accompanied by inflammation and splenomegaly. Our results showed that DDO1002 treatment alleviated aging-induced splenomegaly (Fig. 5D). Flow cytometry analysis of spleen cells showed that the number of B and T cells in the spleen of the DDO1002 group increased, whereas the number of myeloid cells was significantly reduced compared to that in the control group (Fig. 5E and 5F). This indicates that the number of myeloid cells decreased, but the number of lymphoid cells increased. Thus, DDO1002 improved myeloid hematopoiesis in the spleens of aged mice. During aging, the self-renewal ability of HSCs and proliferative ability of hematopoietic progenitor cells are impaired [30]. BM cells from the control and DDO1002 groups were cultured *in vitro*. A colony-forming unit (CFU) assay was performed to determine the effect of DDO1002 on the proliferation and differentiation of BM cells in aged mice. The DDO1002 group showed a significant increase in the number of total CFUs, mainly those generating myeloid cells, compared to the control group (Fig. 5G). To further ascertain the effects of DDO1002 treatment on HSC self-renewal capacity, a competitive transplantation assay was performed. We transplanted freshly isolated BM cells from control and DDO1002-treated aged mice groups (CD45.2) into lethally irradiated recipient mice (CD45.1), with equivalent competing cells (CD45.1) (Fig. 5H). After transplantation, we analyzed the proportion of donor-derived B-, T-, and myeloid cells from the peripheral blood of recipient mice monthly. A higher PB chimerism was observed in DDO1002-treated group, especially in the lymphoid baised cells (Fig. 5I), suggesting that DDO1002 treatment is critical for HSC self-renewal and repopulation capacity in aged mice. Finally, we investigated the effect of DDO1002 on the expression of Nrf2 downstream genes in the BM cells of aged mice using real-time PCR. The expression of Nrf2 downstream genes (HO-1, NQO1, and GCLM) in BM cells of aged mice was significantly upregulated after DDO1002 treatment (Fig. S3H and S3I). These results indicate that DDO1002 alters hematopoiesis in the spleen and enhances hematopoiesis in aged mice by activating Nrf2-mediated antioxidant genes.

### DDO1002 treatment downregulates the ROS pathway and inflammation-related pathways in aged HSCs

**Figure 6.**
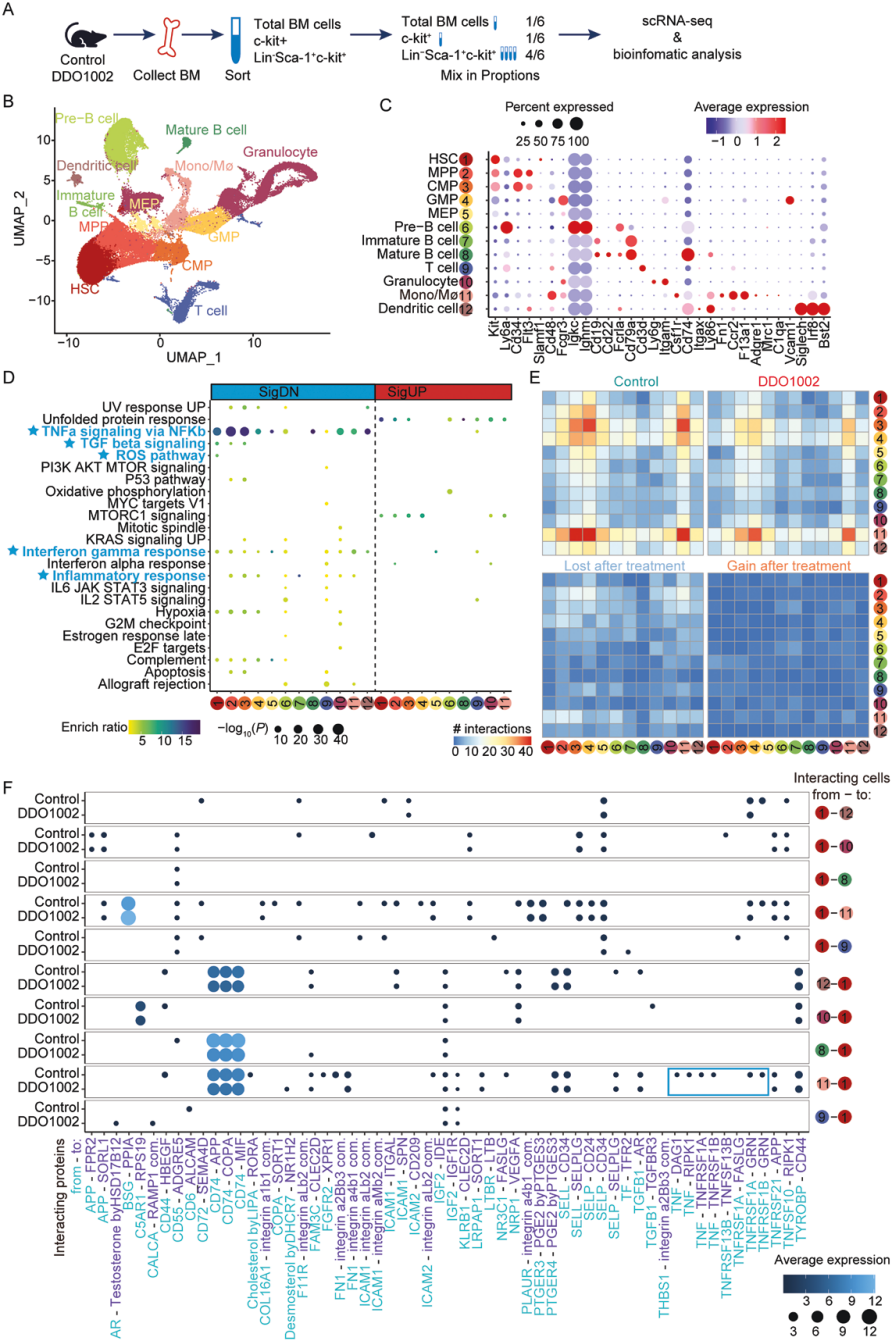
**DDO1002 treatment downregulates the ROS and inflammation-related pathways in aged HSCs.** (A) Flowchart for cell collection and data analysis in scRNA-seq. BM cells were harvested from DDO1002-treated and control mice. Whole BM cells, immature hematopoietic cells (c-kit^+^), and hematopoietic stem and progenitor cells (Lin^−^Sca-1^+^c-kit^+^) were collected by flow cytometric sorting, mixed at a 1:1:4 ratio, and then subjected to 10x Genomics Chromium platform. (B) UMAP plot of scRNA-seq data generated from mixed cells harvested from DDO1002-treated and control mice (*n* = 2 mice), as described in (A). Clusters are differentially colored and labeled according to the dominant population cell identity, which is assigned based on the gene expression signatures in (C). (C) Expression of known markers in each cell type. (D) Dot plot showing hallmarks enriched by up- and downregulated genes in each cell type after treatment with DDO1002. (E) Heatmaps show the number of cellular interactions among all cell types in control (upper left) and DDO1002-treated (upper right) samples and those lost (bottom left) and gained (bottom right) upon DDO1002 treatment. (F) Dot plot shows the cellular interaction pairs between HSCs and immune cells in control and DDO1002-treated samples. "com." stands for complex.

To explore the impact of DDO1002 on hematopoietic regulation in aged mice, single-cell RNA sequencing (scRNA-seq) was performed on BM cells from control and DDO1002-treated mice. Whole BM cells, immature hematopoietic cells (c-kit^+^), and HSCs (Lin^−^c-kit^+^Sca-1^+^) were sorted by flow cytometry and mixed at a ratio of 1:1:4. The combined cells were subjected to scRNA-seq analysis (Fig. 6A). Twenty-three unbiased cell clusters were identified and visualized using uniform manifold approximation and projection (UMAP) (Fig. S4B), spanning from HSCs to mature hematopoietic cells. Cells from control and DDO1002-treated mice were relatively evenly distributed across clusters (Fig. S4A and S4B). Each cluster was defined based on the following canonical marker genes [31, 32]: HSCs (Ly6a^+^c-kit^+^CD34^−^Flt3^−^CD150^+^CD48^−^), multipotent progenitors (MPPs, Ly6a^+^c-kit^+^CD34^+^Flt3^+^CD150^−^CD48^+^), common myeloid progenitors (CMPs, c-kit^+^CD34^+^Ly6a^−^Fcgr3^−^), granulocyte/monocyte progenitors (GMPs, c-kit^+^CD34^+^Ly6a^−^Fcgr3^+^), megakaryocyte/erythroid progenitors (MEPs, c-kit^+^CD34^−^Ly6a^−^), granulocytes (CD11b^+^Ly6g^+^), monocyte/macrophages (CD115^+^Ly86^+^F4/80^+^CD11b^+^Fn1^+^Ccr2^+^F13a^+^Mrc1^+^C1qa^+^Vcalm1^+^), dendritic cells (CD11c^+^CD74^+^Siglech^+^Irf8^+^Bst2^+^), pre-B cells (Igkc^+^Ighm^+^), Immature B cells (CD19^+^CD79a^+^), Mature B cells (CD19^+^CD22^+^Fcrla^+^CD79a^+^), and T cells (CD3d^+^) (Fig. 6B, 6C and S4C).

To further explore the underlying mechanism, we examined changes in the transcriptomics of each cell type and identified hundreds of differentially expressed genes (Fig. S5A). Notably, downregulated genes were enriched in TNFα signaling via NF-κB in almost all cell types of DDO1002-mice compared to those in control mice (Fig. 6D). Furthermore, gene set enrichment analysis of gene expression changes in DDO1002-treated mice revealed several downregulated hallmarks of inflammation, including TGF-β signaling, interferon gamma response, and inflammatory response (Fig. 6D). Notably, the ROS pathway was downregulated in the HSCs of DDO1002-treated mice compared to control mice (Fig. 6D), suggesting that DDO1002 could improve HSC function. To delineate the overall changes within the bone marrow microenvironment, the analysis of cellular interactions was performed using CellphoneDB (Fig. 6E). Interestingly, we found that more interactions were lost after DDO1002 treatment compared with the interactions gained. The most lost interactions occurred between monocyte/macrophages and HSC/MPP/CMPs and among HSPCs (Fig. 6E). We further looked into the interacting gene pairs between immune cells and HSCs and found that several interactions involving TNF or its receptors were lost between HSCs and monocyte/macrophages (Fig. 6F), which again suggested decreased activity of TNFa signaling and improved immune microenvironment.

HSCs displayed both upregulated and downregulated pathways and key genes after DDO1002 treatment. The proliferation-associated genes were up-regulated in HSCs (Fig. S5B); however, aged HSC signature were down-regulated after DDO1002 treatment (Fig. S5C). Moreover, several genes associated HSC quiescence/stemness were up-regulated (Fig. S5E). These data suggested a functional recovery of HSCs upon DDO1002 treatment. DNA repair genes were down-regulated, which may be a consequence of less DNA damage in the more “healthy” HSCs (Fig. S5D). Identified downregulated pathways and key genes were displayed including the interferon gamma response (*Ccl5, Ptgs2, Tnfaip2, Nfkbia, Irf1*), ROS pathway (*Junb, Ftl1, Lsp1, Prdx6*), and TNFA signaling via NF-κB (*Hes1, Nr4a1, Efr3, PIK2*), whereas identified upregulated pathways and key genes included the unfolded protein response (*Xbp1, Edem1, Hsp90b1, Herpud1, Spcs1, Pdia6*) and MTORC1 signaling (*Sdf2l1, Vldlr*) (Fig. S5F).

## Discussion

**Figure 7.**
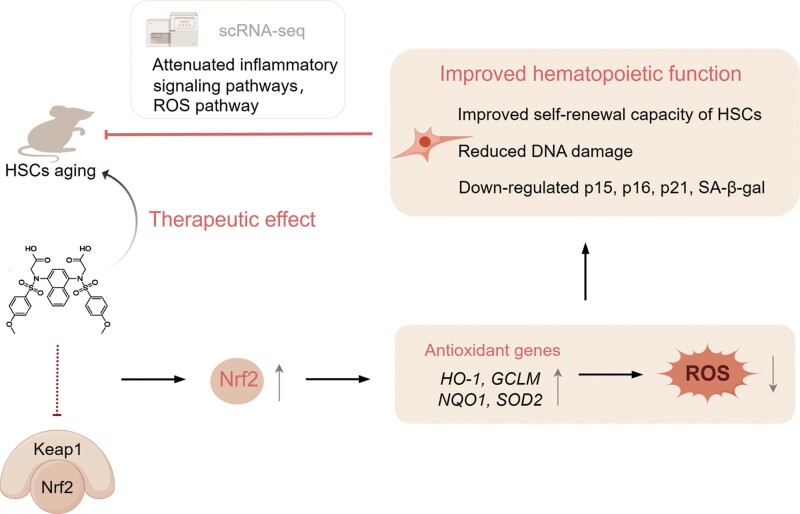
**Constructive model describing the role of DDO1002 in improving hMSC senescence hematopoietic stem cell aging and stress response.** DDO1002 can disturb the binding of Keap1 and Nrf2 to upregulate the expression of Nrf2, which in turn activates the expression of Nrf2-driven antioxidant genes. This activation can diminish intracellular levels of ROS, thereby attenuating cellular senescence. Furthermore, DDO1002 can ameliorate stress-induced DNA damage and improve the reconstitution and self-renewal capacity of HSCs in aged mice. Single-cell sequencing analysis revealed that DDO1002 treatment attenuated intracellular inflammatory signaling pathways and mitigated ROS pathway in aged HSCs. Therefore, DDO1002 is a potential therapeutic agent to ameliorating impaired hematopoiesis (this figure was partially created with Figdraw).

DDO1002, an effective KEAP1–NRF2 PPI inhibitor, activates the expression of NRF2-mediated cytoprotective genes at the mRNA level [17]. However, the therapeutic effects of DDO1002 on hematopoietic injury have not yet been studied. Here, we showed that DDO1002 upregulates Nrf2, activates the ARE pathway, stimulates the expression of antioxidant genes at the protein level, and delays cellular senescence (Fig. 7). Indeed, NRF2 is a REDOX sensor that regulates the expression of more than 250 genes, including antioxidant, cytoprotective, and detoxifying enzymes, thereby counteracting ROS and playing an important role in protecting against aging and age-related diseases [33, 34]. Moreover, NRF2 activation could improve REDOX homeostasis in MSCs to enhance cellular genomic stability, thereby preventing accelerated stem cell depletion [8].

To explore the effects of DDO1002 on the hematopoietic system further, we used an irradiation-induced hematopoietic injury model. Ionizing radiation at a dose of 4 Gy was used to induce hematopoietic injury. The number of long-term HSCs and lymphoid-primed MPP4 cells were increased by DDO1002 treatment, as did the number of B cells in peripheral blood. Therefore, we concluded that DDO1002 alleviates hematopoietic injury. Several natural compounds have been reported to alleviate hematopoietic injury by activating Nrf2. For example, theaflavins, 3,3ʹ-Diindolylmethane, and a resveratrol dimer (Vam3) can reduce oxidative stress levels in HSCs through the Nrf2 pathway, alleviate TBI-induced HSC injury, and enhance HSC function [23, 27, 35]. However, the detailed targets of these compounds and their underlying mechanisms remain unclear. In addition, some compounds, including monomethyl fumarate and sulforaphane, reportedly alleviate the development of chronic diseases, such as diabetes, by activating Nrf2 through binding to the cysteine of Keap1 [13]. However, because these activators are electrophilic compounds, cysteine residues on proteins other than KEAP1 may also be modified. DDO1002, a KEAP1–NRF2 PPI inhibitor, is designed according to the interaction interface of KEAP1–NRF2, which displays more specific targeting. Therefore, we focused on DDO1002 with high affinity for KEAP1. It has a median effect concentration of 28.6 nM [17], indicating its safety and effectiveness.

Considering that NRF2 is involved in aging and hematopoietic injury, increased ROS levels are observed in the HSCs of aging mice, as well as increased DNA damage and cell apoptosis, leading to HSC dysfunction [1, 36]. Here, we examined the effects of DDO1002 on the hematopoietic system and HSCs in naturally aging (20-month-old) mice. DDO1002 significantly increased the number of red blood cells, hemoglobin, average concentration of hemoglobin in red blood cells, and percentage of monocytes in the peripheral blood of aged mice. Extramedullary hematopoiesis was affected that the proportions of B and T cells in the spleen increased while the proportion of myeloid cells was decreased. Moreover, the colony-forming ability of BM cells was enhanced, and antioxidant genes were upregulated. We then used scRNA-seq to explore whether DDO1002 treatment affected HSC function in aged mice. The results showed that DDO1002 treatment in mice attenuated intracellular inflammatory signaling in BM cells and reduced the ROS pathway in aged HSCs, suggesting that this inhibitor may restore the viability of aged HSCs. This is consistent with previous studies suggesting that Nrf2 upregulation can reduce ROS levels and oxidative and inflammatory stress [37–40], and therefore represents an important approach for the prevention of cancer and other chronic diseases. Diverse inflammation-related genes were downregulated by the treatment of DDO1002, such as *Ccl5*, a pro-inflammatory chemokine, is essential for tissue homing of inflammatory cells and may be a potential target for inflammation disorders [41]. *Tnfaip2* is involved in inflammation disease, immune response, and hematopoiesis, and it has been reported that *Tnfaip2* can promote atherogenesis through regulating macrophages inflammation [42]. *Nfkbia* is associated with pro-inflammatory response and immune regulation [43]. *Irf1* also plays a vital role in regulating downstream inflammation and cell death [44]. This further suggests that DDO1002 treatment can alleviate cellular inflammation in HSCs of aged mice.

Moreover, inflammatory stress attenuates hematopoietic reconstitution via the destruction of stromal niche, leading to poor graft function (PGF) after hematopoietic stem cell transplantation (HSCT). Bone marrow-derived mesenchymal stem cells (BMSCs) from PGF patients have high ROS levels and impaired self-renewal and HSC-supporting function. All-trans retinoic acid treatment or inflammatory stress up-regulates the expression of RIG-I in BMSCs to promote the degradation of Nrf2, thereby increasing the level of ROS, leading to BMSCs damage and finally destroying their function in supporting hematopoietic reconstitution [45]. Antioxidant therapy with DDO1002 may reduce inflammatory stress and restore the homeostasis of BMSCs, thereby increasing the engraftment of HSCT.

In both irradiation-induced hematopoietic injury and aging models, DDO1002 could up-regulate Nrf2 to activate the antioxidant pathway. However, the underlying molecular mechanisms of DDO1002 in these two models might be different. In TBI-induced hematopoietic injury, DDO1002 can alleviate cellular damage, reduce ROS level, and increase the number of hematopoietic stem cells and lymphocyte-biased multipotent progenitor cells. In naturally aging mice, the administration of DDO1002 could down-regulate the ROS pathway and inflammation-related pathways in HSCs, and alter the function of HSCs (Fig. 7).

In summary, our findings demonstrated that DDO1002, a NRF2–KEAP1 PPI inhibitor, can effectively alleviate radiation-induced hematopoietic injury and enhance the function of hematopoietic stem/progenitor cells in aged mice. Therefore, this NRF2–KEAP1 PPI inhibitor may be useful for treating hematopoietic dysfunction induced by ionizing radiation and aging.

## Research limitations

Despite revealing that DDO1002 delayed cell senescence and ameliorated hematopoietic impairment through the activation of the NRF2–ARE signaling pathway, this study had some limitations. First, the exact mechanism requires further elucidation, such as the targeting genes of pathways affected in hematopoietic stem/progenitor of DDO1002-treated aging mice need further verification. In addition, the long-term safety and efficacy of DDO1002, as well as its systemic impact on various tissues, require further exploration.

## Methods

### Research ethics

The mice used in this study were raised under SPF conditions. All experiments on mice were approved by the Animal Ethics Committee of Hangzhou Normal University. The corresponding ethics approval number is HSD-20231214-01.

### Cell culture and DDO1002 administration

The hMSCs used in this study were cultured in MSC BM basal medium (DAKEWE, China). To determine the effect of DDO1002 on senescent hMSCs, hMSCs (P25) were treated with 1, 2.5, or 5 µM DDO1002 for 36 h. To determine the effect of DDO1002 on H_2_O_2_-induced senescent cells, hMSCs were treated with 150 µM H_2_O_2_ for 3 h and then with 1, 2.5, and 5 µM DDO1002 for more than 24 h. All cells were incubated at 37°C in a humidified incubator with 5% CO_2_.

### Mice

Four-month-old C57BL/6 mice were purchased from the Animal Experiment Centre of Hangzhou Normal University and randomly divided into irradiation and irradiation + DDO1002 groups. All mice received TBI with 4 Gy of R-rays at a dose rate of 0.407 Gy/min. Mice in the irradiation + DDO1002 group received 50 mg/kg DDO1002 solution (5% DMSO, 30% PEG300, 10% Tween 80, and 55% ddH_2_O) by intraperitoneal injection every 2 days for 54 days, starting 6 days after irradiation. Mice in the irradiation + control group received the same volume of solvent control for the same duration and frequency as mice in the irradiation + DDO1002 group. The mice were sacrificed 70 days after TBI.

For aging, 22-month-old mice were randomly divided into control and DDO1002 groups. The DDO1002 group received 50 mg/kg of DDO1002 solution intraperitoneally every other day for 60 days. Mice in the control group received the same volume of solvent. All mice were housed in the Animal Management Center of Hangzhou Normal University under condition of natural light, food, and water. All procedures were approved by the Animal Ethics Committee of Hangzhou Normal University.

### Flow cytometry analysis

To obtain the percentage of B cells, myeloid cells, and T cells, 20 μL of peripheral blood was collected and the red blood cells were removed (BD FACS Lysing Solution). Then, FITC-conjugated anti-CD3, FITC-conjugated anti-B220, APC-conjugated anti-B220, and APC-conjugated anti-CD11b antibodies were used at 4°C. BM cells were isolated and filtered for HSC analysis. Approximately 10^7^ cells were stained for with a biotin-conjugated lineage cocktail (anti-CD11B, anti-CD4, anti-B220, anti-CD8, anti-Ter119, and anti-Gr-1 antibodies) alongside PE-Cy7-conjugated anti-Sca-1, PE-conjugated CD135, APC-conjugated anti-c-kit, BV605-conjugated anti-CD150, BV510-conjugated anti-CD48 antibodies. These cells were further stained with APC-Cy7-conjugated streptavidin before analysis. The LSRFortessa Cell Analyzer (BD Biosciences) was used to analyze the prepared samples. For transplantation analysis, PE-conjugated anti-CD45.2, Percp-Cy5.5-CD45.1, and antibodies utilized in PB analysis were adopted to evaluate donor-derived B-, T-, and myeloid cells. The LSRFortessa Cell Analyzer (BD Biosciences) was used to analyze the prepared samples.

### Colony of granulocyte macrophage cells (CFU-GM)

Approximately 10^4^ BM cells from the control and DDO1002 groups of aging mice were cultured in M3534 methylcellulose medium for 7 days. Then, we counted the CFU values for granulocyte macrophage cells and granulocyte, erythrocyte, monocyte, and megakaryocyte cells, as well as burst-forming unit–erythroid colonies.

### Competitive transplantation assays

BM cells were freshly acquired from control and DDO1002-treated aged mice groups (CD45.2^+^). Subsequently, 1 × 10^6^ cells were isolated and mixed with an equal amount of BM cells from competitor mice (CD45.1^+^) and transplanted into recipient mice (CD45.1^+^) by tail vein injection. Before transplantation, the recipient mice were irradiated with a fatal dose (8.3 Gy). Antibiotic-treated water was given to recipient mice for 3 weeks after transplantation.

### Senescence assay

A β-galactosidase assay kit (Beyotime, China) was utilized to quantify the β-galactosidase activity in cells according to the manufacturer’s guidelines. Blue-stained cells were considered positive (senescent cells). Ten regions were randomly selected to calculate the percentage of aged cells using an optical microscope (Nikon, Tokyo, Japan).

### Quantitative real-time PCR

Total RNA was extracted from cells (hMSCs and BM cells) with TRIzol reagent (Thermo Fisher Scientific, USA). Reverse transcription was performed using a commercial kit (Vazyme) to reverse transcribe total RNA into cDNA for quantitative real-time PCR (qPCR). Subsequently, cDNA dilutions were used for quantitative PCR using the SYBR Select Master Mix for CFX (Vazyme, City, China) in a Bio-Rad CFX Real-Time PCR system (Bio-Rad, USA). GAPDH expression levels were then normalized. The primer sequences are listed in Table S1.

### Detection of ROS levels

Three groups of hMSCs were treated with DMSO, H_2_O_2_, and H_2_O_2_ + DDO1002. Then, the cells were harvested and incubated with 10 μM 2,7-dichlorodihydrofluorescein diacetate (Beyotime Biotechnology) for 20 min at 37°C. For aging mice, 5 × 10^6^ BM cells stained with LSK antibodies were incubated with 10 μM 2,7-dichlorodihydrofluorescein diacetate (Beyotime Biotechnology) for 20 min at 37°C. Intracellular ROS levels were evaluated by measuring the mean fluorescence intensity of 2,7-dichlorodihydrofluorescein diacetate using flow cytometry.

### Western blot analysis

Cells were collected and lysed in ice-cold RIPA lysis buffer (Beyotime) supplemented with PMSF (Beyotime). The total protein was extracted, electrophoresed, and transferred to PVDF membranes. Membranes were incubated with primary antibodies at 4°C. The primary antibodies utilized in this study were against the following: NRF2 (1:500, ER1706-41, Huabio), HO-1(1:1000, ER1802-73, Huabio), GCLM (ET1705-87), NQO1 (ET1702-50), SOD2(ET-1701-54), γH2AX (1:5000, ab81299, Abcam), p16INK4a (1:1000, ab270058, Abcam), p21(1:1500, ab109520, Abcam), and β-actin (1:2000, HA601037, Huabio). Membranes were developed using an ECL substrate kit (Vazyme), visualized using an Imaging System, and quantified using the ImageJ software.

### RNA-seq assays

Total RNA was purified from cells using TRIzol reagent following the standard protocol. Prior to library preparation, RNA integrity was evaluated using an RNA Nano 6000 Assay Kit on a Bioanalyzer 2100 system (Agilent Technologies, USA). mRNA was selected from the total RNA before the synthesis of first-/second-strand cDNAs. Following adenylation of the 3ʹ ends of DNA fragments, an adaptor with a hairpin loop structure was added for hybridization. Subsequently, PCR was performed to obtain 370–420 bp cDNA fragments. Finally, library quality was evaluated using an Agilent Bioanalyzer 2100 system. For the RNA-seq data, an alignment-free approach was adopted using Kallisto (version 0.46) to quantify transcript abundance. Differential expression analyses were performed using DESeq2 (version 1.42.0).

### scRNA-seq and bioinformatic analysis

#### scRNA-seq library preparation and sequencing

The whole BM cell, immature hematopoietic cells (c-kit^+^), and hematopoietic stem cells (Lin^−^c-kit^+^sca-1^+^) were sorted using flow cytometry and mixed at a ratio of 1:1:4. The mixed cells in suspensions were loaded onto microfluidic devices and scRNA-seq libraries were constructed according to the Singleron GEXSCOPE™ protocol using the GEXSCOPE™ Single-Cell RNA Library Kit (Singleron, 1110011). After quality checks, libraries were sequenced on an Illumina NovaSeq with 150 bp paired end reads.

#### scRNA-seq analysis

Raw reads were mapped to mouse genome mm10 to generate gene expression matrix using CeleScope (v2.0.7) with default parameters. In total, 29,277 and 30,715 cells were detected for control and DDO1002-treated mice, respectively. Subsequent analysis were performed using R package Seurat (v4.1.1). In details, genes detected in less than 3 cells were excluded, and cells were required to have > 200 and < 5000 genes, < 10% reads mapping to mitochondrial genes, and < 5% of mapped reads mapping to hemoglobin genes. After filtering, 29,129 and 30,469 cells were retained for control and DDO1002-treated mice, respectively. Principal component analysis (PCA) was performed on the scaled integrated data, and, using the first 30 dimensions, dimensionality reduction was performed using UMAP and 23 cluster were found with resolution of 0.5. Clusters were annotated on the basis of expression of canonical markers. Differential expression analysis in DDO1002-treated versus control mice was performed by “FindMarkers” function based on Wilcox likelihood-ratio test. Significantly differentially expressed genes upon DDO1002 treatment were identified by selecting genes expressed in more than 10% of the cells and with |avg_log_2_FC| > 0.25 and p_val_adj < 0.05. Gene Set Enrichment Analysis (GSEA) were performed using clusterProfiler (v4.2.2). The number of DEGs and enrichment in hallmarks were visualized by ggplot2 (v3.4.1), and the network between DEGs and enriched hallmarks was visualized by Cytoscape (v3.9.1). Cellular interactions were analyzed using CellphoneDB (v4.0.0).

### Statistical analysis

All statistical analyses were performed using GraphPad Prism 8. Statistical significance between the two groups was determined using an unpaired two-tailed Student’s *t*-test. Group analyses were performed using one- or two-way analysis of variance (ANOVA). The level of statistical significance was set at *P* < 0.05. Data were presented as the mean ± SD or mean ± SEM as indicated in the figure legends.

## Supplementary Material

lnae043_suppl_Supplementary_Figures_S1-S5_Tables_S1

## Data Availability

The scRNA-seq data generated in this study has been deposited in the GEO database under accession code GSE275533.

## References

[1] Liang Y , Van Zant G, Szilvassy SJ. Effects of aging on the homing and engraftment of murine hematopoietic stem and progenitor cells. Blood 2005;106:1479–87. 15827136 10.1182/blood-2004-11-4282 PMC1895199

[2] Ghaffari S. Oxidative stress in the regulation of normal and neoplastic hematopoiesis. Antioxid Redox Signal 2008;10:1923–40. 18707226 10.1089/ars.2008.2142 PMC2932538

[3] McMahon M , Thomas N, Itoh K, et al Redox-regulated turnover of Nrf2 is determined by at least two separate protein domains, the redox-sensitive Neh2 degron and the redox-insensitive Neh6 degron. J Biol Chem 2004;279:31556–67. 15143058 10.1074/jbc.M403061200

[4] Hayes JD , Dinkova-Kostova AT. The Nrf2 regulatory network provides an interface between redox and intermediary metabolism. Trends Biochem Sci 2014;39:199–218. 24647116 10.1016/j.tibs.2014.02.002

[5] Itoh K , Mimura J, Yamamoto M. Discovery of the negative regulator of Nrf2, Keap1: a historical overview. Antioxid Redox Signal 2010;13:1665–78. 20446768 10.1089/ars.2010.3222

[6] Wang H , Liu K, Geng M, et al RXRα inhibits the NRF2-ARE signaling pathway through a direct interaction with the Neh7 domain of NRF2. Cancer Res 2013;73:3097–108. 23612120 10.1158/0008-5472.CAN-12-3386

[7] Modi R , McKee N, Zhang N, et al Stapled peptides as direct inhibitors of Nrf2-sMAF transcription factors. J Med Chem 2023;66:6184–92. 37097833 10.1021/acs.jmedchem.2c02037 PMC10184664

[8] Kubben N , Zhang W, Wang L, et al Repression of the antioxidant NRF2 pathway in premature aging. Cell 2016;165:1361–74. 27259148 10.1016/j.cell.2016.05.017 PMC4893198

[9] Song G , Wang J, Liu J, et al Dimethyl fumarate ameliorates erectile dysfunction in bilateral cavernous nerve injury rats by inhibiting oxidative stress and NLRP3 inflammasome-mediated pyroptosis of nerve via activation of Nrf2/HO-1 signaling pathway. Redox Biol 2023;68:102938. 37931471 10.1016/j.redox.2023.102938 PMC10652210

[10] Zhang DD , Hannink M. Distinct cysteine residues in Keap1 are required for Keap1-dependent ubiquitination of Nrf2 and for stabilization of Nrf2 by chemopreventive agents and oxidative stress. Mol Cell Biol 2003;23:8137–51. 14585973 10.1128/MCB.23.22.8137-8151.2003 PMC262403

[11] Cho H , Hartsock MJ, Xu Z, et al Monomethyl fumarate promotes Nrf2-dependent neuroprotection in retinal ischemia-reperfusion. J Neuroinflammation 2015;12:239. 26689280 10.1186/s12974-015-0452-z PMC4687295

[12] Yan N , Xu Z, Qu C, et al Dimethyl fumarate improves cognitive deficits in chronic cerebral hypoperfusion rats by alleviating inflammation, oxidative stress, and ferroptosis via NRF2/ARE/NF-κB signal pathway. Int Immunopharmacol 2021;98:107844. 34153667 10.1016/j.intimp.2021.107844

[13] Mohamadi N , Baradaran Rahimi V, Fadaei MR, et al A mechanistic overview of sulforaphane and its derivatives application in diabetes and its complications. Inflammopharmacol 2023;31:2885–99. 10.1007/s10787-023-01373-z 37955784

[14] Reisman SA , Chertow GM, Hebbar S, et al Bardoxolone methyl decreases megalin and activates Nrf2 in the kidney. J Am Soc Nephrol 2012;23:1663–73. 22859857 10.1681/ASN.2012050457 PMC3458470

[15] Rizk DV , Silva AL, Pergola PE, et al Effects of bardoxolone methyl on magnesium in patients with Type 2 diabetes mellitus and chronic kidney disease. Cardiorenal Med 2019;9:316–25. 31170712 10.1159/000500612 PMC6751480

[16] Fukutomi T , Takagi K, Mizushima T, et al Kinetic, thermodynamic, and structural characterizations of the association between Nrf2-DLGex degron and Keap1. Mol Cell Biol 2014;34:832–46. 24366543 10.1128/MCB.01191-13 PMC4023822

[17] Jiang ZY , Lu M-C, Xu LL, et al Discovery of potent Keap1-Nrf2 protein-protein interaction inhibitor based on molecular binding determinants analysis. J Med Chem 2014;57:2736–45. 24512214 10.1021/jm5000529

[18] Sun Y , Huang J, Chen Y, et al Direct inhibition of Keap1-Nrf2 protein-protein interaction as a potential therapeutic strategy for Alzheimer’s disease. Bioorg Chem 2020;103:104172. 32890991 10.1016/j.bioorg.2020.104172

[19] Lu MC , Zhao J, Liu Y-T, et al CPUY192018, a potent inhibitor of the Keap1-Nrf2 protein-protein interaction, alleviates renal inflammation in mice by restricting oxidative stress and NF-κB activation. Redox Biol 2019;26:101266. 31279986 10.1016/j.redox.2019.101266 PMC6614503

[20] Merchant AA , Singh A, Matsui W, et al The redox-sensitive transcription factor Nrf2 regulates murine hematopoietic stem cell survival independently of ROS levels. Blood 2011;118:6572–9. 22039262 10.1182/blood-2011-05-355362 PMC3242719

[21] Tsai JJ , Dudakov JA, Takahashi K, et al Nrf2 regulates haematopoietic stem cell function. Nat Cell Biol 2013;15:309–16. 23434824 10.1038/ncb2699 PMC3699879

[22] Hu L , Zhang Y, Miao W, et al Reactive oxygen species and Nrf2: functional and transcriptional regulators of hematopoiesis. Oxid Med Cell Longev 2019;2019:5153268. 31827678 10.1155/2019/5153268 PMC6885799

[23] Zhang J , Xue X, Han X, et al Vam3 ameliorates total body irradiation-induced hematopoietic system injury partly by regulating the expression of Nrf2-targeted genes. Free Radic Biol Med 2016;101:455–64. 27989754 10.1016/j.freeradbiomed.2016.10.501

[24] Qiu X , Brown K, Hirschey MD, et al Calorie restriction reduces oxidative stress by SIRT3-mediated SOD2 activation. Cell Metab 2010;12:662–7. 21109198 10.1016/j.cmet.2010.11.015

[25] Wang W , Zheng Y, Sun S, et al A genome-wide CRISPR-based screen identifies KAT7 as a driver of cellular senescence. Sci Transl Med 2021;13:eabd2655. 33408182 10.1126/scitranslmed.abd2655

[26] Li W , Wang X, Dong Y, et al Nicotinamide riboside intervention alleviates hematopoietic system injury of ionizing radiation-induced premature aging mice. Aging Cell 2023;22:e13976. 37650560 10.1111/acel.13976 PMC10652312

[27] Han X , Zhang J, Xue X, et al Theaflavin ameliorates ionizing radiation-induced hematopoietic injury via the NRF2 pathway. Free Radic Biol Med 2017;113:59–70. 28939421 10.1016/j.freeradbiomed.2017.09.014

[28] Santivasi WL , Xia F. Ionizing radiation-induced DNA damage, response, and repair. Antioxid Redox Signal 2014;21:251–9. 24180216 10.1089/ars.2013.5668

[29] Xu G , Wu H, Zhang J, et al Metformin ameliorates ionizing irradiation-induced long-term hematopoietic stem cell injury in mice. Free Radic Biol Med 2015;87:15–25. 26086617 10.1016/j.freeradbiomed.2015.05.045 PMC4707049

[30] de Haan G , Lazare SS. Aging of hematopoietic stem cells. Blood 2018;131:479–87. 29141947 10.1182/blood-2017-06-746412

[31] Zeng X , Li X, Li X, et al Fecal microbiota transplantation from young mice rejuvenates aged hematopoietic stem cells by suppressing inflammation. Blood 2023;141:1691–707. 36638348 10.1182/blood.2022017514 PMC10646769

[32] Zhong J , Mao X, Li H, et al Single-cell RNA sequencing analysis reveals the relationship of bone marrow and osteopenia in STZ-induced type 1 diabetic mice. J Adv Res 2022;41:145–58. 36328744 10.1016/j.jare.2022.01.006 PMC9637485

[33] Silva-Palacios A , Ostolga-Chavarría M, Zazueta C, et al Nrf2: molecular and epigenetic regulation during aging. Ageing Res Rev 2018;47:31–40. 29913211 10.1016/j.arr.2018.06.003

[34] O’Connell MA , Hayes JD. The Keap1/Nrf2 pathway in health and disease: from the bench to the clinic. Biochem Soc Trans 2015;43:687–9. 26551713 10.1042/BST20150069

[35] Lu L , Dong J, Li D, et al 3,3’-diindolylmethane mitigates total body irradiation-induced hematopoietic injury in mice. Free Radic Biol Med 2016;99:463–71. 27609226 10.1016/j.freeradbiomed.2016.09.007

[36] Pang WW , Price EA, Sahoo D, et al Human bone marrow hematopoietic stem cells are increased in frequency and myeloid-biased with age. Proc Natl Acad Sci USA 2011;108:20012–7. 22123971 10.1073/pnas.1116110108 PMC3250139

[37] Kobayashi EH , Suzuki T, Funayama R, et al Nrf2 suppresses macrophage inflammatory response by blocking proinflammatory cytokine transcription. Nat Commun 2016;7:11624. 27211851 10.1038/ncomms11624 PMC4879264

[38] El-Shitany NA , Eid BG. Icariin modulates carrageenan-induced acute inflammation through HO-1/Nrf2 and NF-kB signaling pathways. Biomed Pharmacother 2019;120:109567. 31670031 10.1016/j.biopha.2019.109567

[39] Huang JC , Yue Z-P, Yu H-F, et al TAZ ameliorates the microglia-mediated inflammatory response via the Nrf2-ROS-NF-κB pathway. Mol Ther Nucleic Acids 2022;28:435–49. 35505966 10.1016/j.omtn.2022.03.025 PMC9043866

[40] Shimizu R , Hirano I, Hasegawa A, et al Nrf2 alleviates spaceflight-induced immunosuppression and thrombotic microangiopathy in mice. Commun Biol 2023;6:875. 37626149 10.1038/s42003-023-05251-w PMC10457343

[41] Mikolajczyk TP , Szczepaniak P, Vidler F, et al Role of inflammatory chemokines in hypertension. Pharmacol Ther 2021;223:107799. 33359600 10.1016/j.pharmthera.2020.107799

[42] Jin G , Liu Y, Xu W, et al Tnfaip2 promotes atherogenesis by enhancing oxidative stress induced inflammation. Mol Immunol 2022;151:41–51. 36084515 10.1016/j.molimm.2022.08.019

[43] Zhang Y , Gao Y, Jiang Y, et al Histone demethylase KDM5B licenses macrophage-mediated inflammatory responses by repressing Nfkbia transcription. Cell Death Differ 2023;30:1279–92. 36914768 10.1038/s41418-023-01136-x PMC10154333

[44] Sharma BR , Karki R, Rajesh Y, et al Immune regulator IRF1 contributes to ZBP1-, AIM2-, RIPK1-, and NLRP12-PANoptosome activation and inflammatory cell death (PANoptosis). J Biol Chem 2023;299:105141. 37557956 10.1016/j.jbc.2023.105141 PMC10494469

[45] Lou Q , Jiang K, Xu Q, et al The RIG-I-NRF2 axis regulates the mesenchymal stromal niche for bone marrow transplantation. Blood 2022;139:3204–21. 35259210 10.1182/blood.2021013048

