# Supplementary material for: DDO1002, an NRF2–KEAP1 inhibitor, improves hematopoietic stem cell aging and stress response
Source: Life Med. 2024 Dec 12;3(6):lnae043. doi: 10.1093/lifemedi/lnae043 (PMC11748272; doi:10.1093/lifemedi/lnae043)
Supplement: lnae043_suppl_Supplementary_Figures_S1-S5_Tables_S1 [file lnae043_suppl_supplementary_figures_s1-s5_tables_s1.pdf]

Supplementary information

DDO1002, an NRF2–KEAP1 inhibitor, improves haematopoietic stem cell aging and stress response

Yuwen Li, Aiwei Wu, Xinrong Jin, Haiping Shen, Chenyan Zhao, Xiao Yi, Hui Nie, Mingwei Wang, Shouchun Yin, Hongna Zuo, Zhenyu Ju, Zhenyu Jiang, Hu Wang

Supplementary Figure 1-5

Table S1

Supplementary Figures and Figure lengends

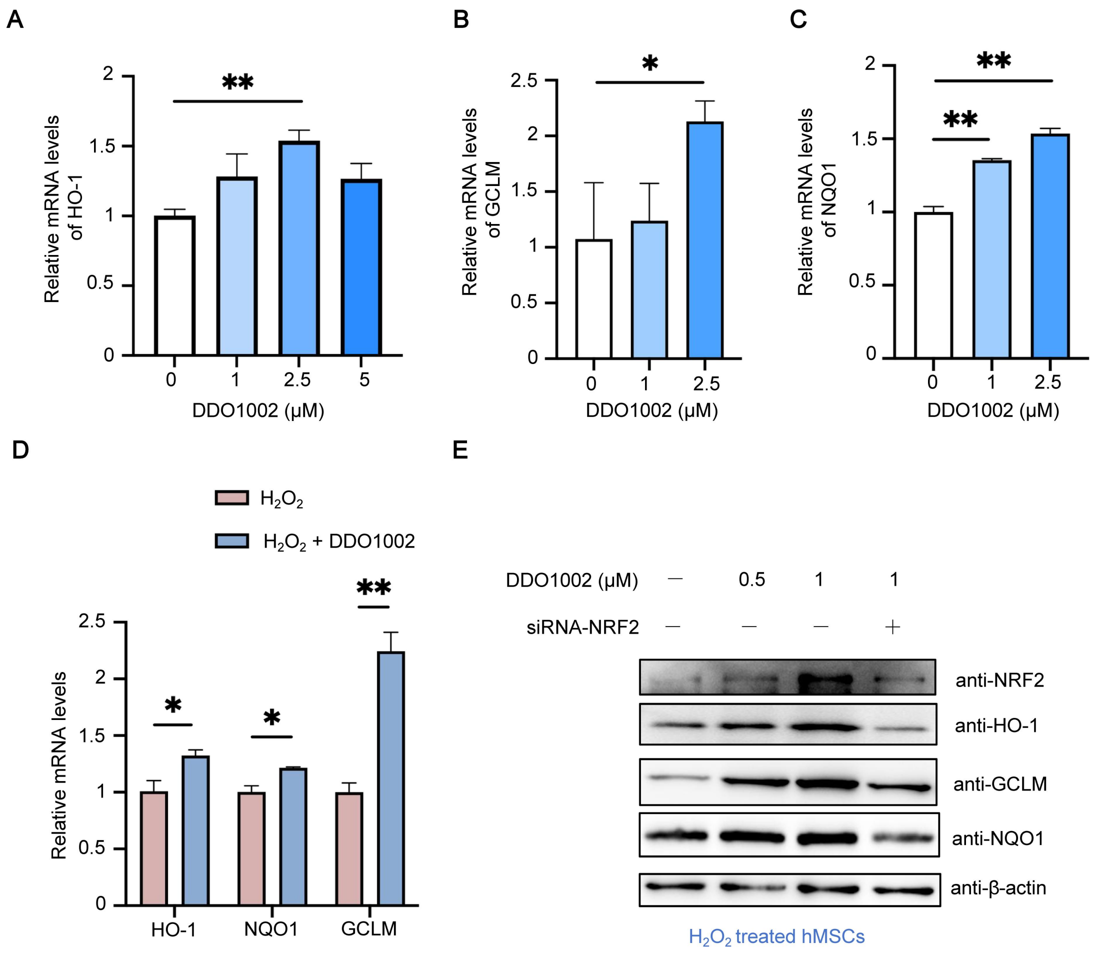

Supplementary Figure 1. DDO1002 activates the ARE pathway by upregulating NRF2.

(A) Relative mRNA levels of HO-1 in hMSCs after treatment with DDO1002 (0, 1, 2.5, and 5 μM) (***P* < 0.01 vs. control).

(B) Relative mRNA levels of GCLM in hMSCs after treatment with DDO1002 (0, 1, 2.5, and 5 μM) (**P* < 0.05 vs. control).

(C) Relative mRNA levels of NQO1 in hMSCs after treatment with DDO1002 (0, 1, 2.5, and 5 μM) (**P* < 0.05 vs. control).

(D) In H_2O_2-induced senescent hMSCs, the relative mRNA levels of HO-1, GCLM, and NQO1 after treatment with DDO1002 (1 μM) (**P* < 0.05, ***P* < 0.01 vs.

control).

(E) Western blot analysis of NRF2, HO-1, NQO-1, and GCLM in senescent hMSCs after NRF2 knockdown by siRNA.

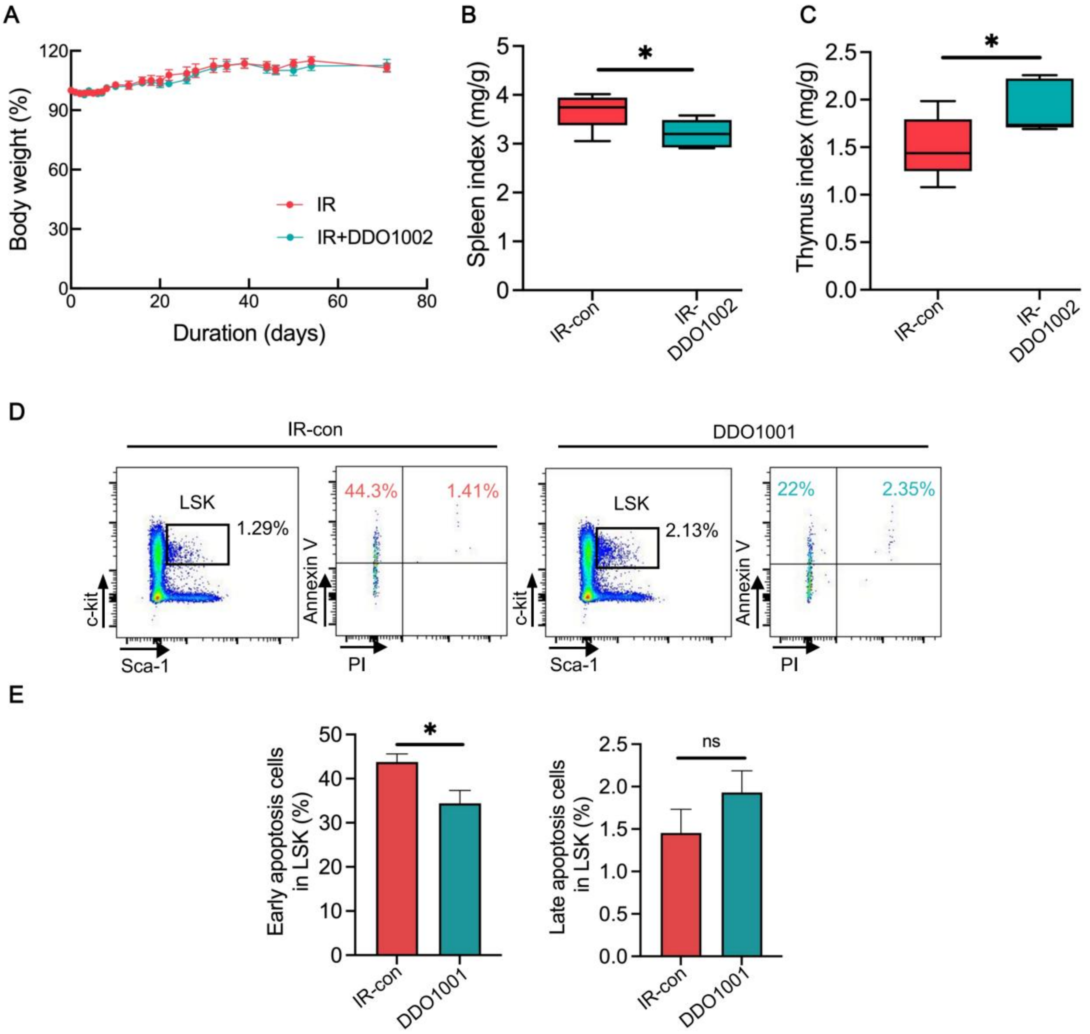

Supplementary Figure 2. DDO1002 can improve TBI-induced hematopoietic injury.

(A) Percentage of body weight was recorded and calculated (initial body weight was 100%).

(B, C) Spleen index (ratio of spleen body weight) and thymus index (ratio of thymus to body weight) of IR-control and IR-DDO1002-treated mice.

(D) FACS analysis of LSK cell apoptosis in IR-control and IR-DDO1002-treated

mice.

(E) Quantitative analysis of early and late apoptotic cells in LSKs from IR-control and IR-DDO1002-treated mice.

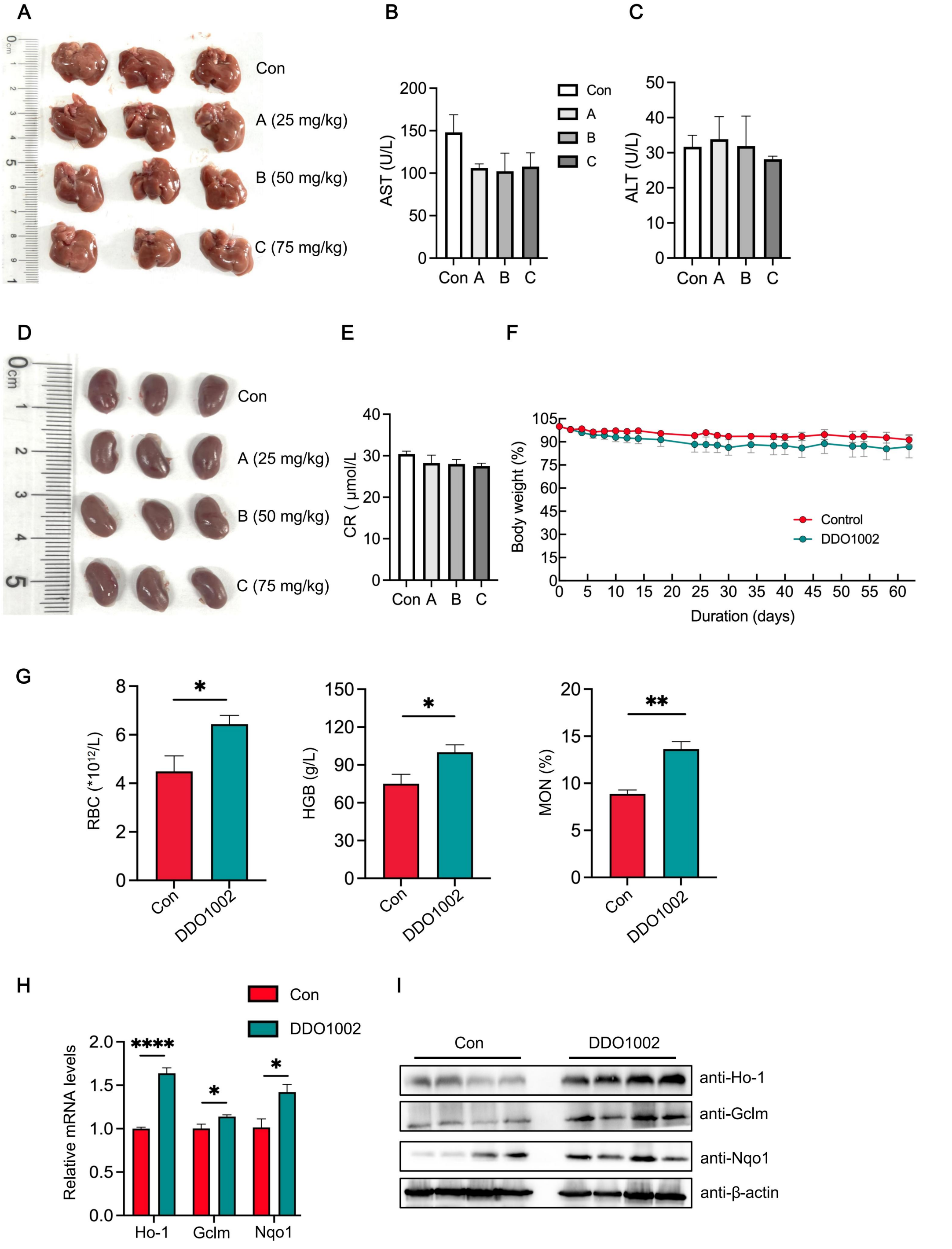

Supplementary Figure 3. DDO1002 treatment has no adverse effects and it can improve haematopoiesis in aged mice.

(A) Images showing the morphology of liver in the control, A (25 mg/kg dose of DD01002), B (50 mg/kg dose of DD01002) and C (75 mg/kg dose of DD01002 groups, ($n \geq 3$).

(B,C) Serum levels of aspartate aminotransferase (AST) and Serum alanine transaminase (ALT) in control and DDO1002-treated mice, ($n \geq 3$).

(D) Images showing the morphology of kidney in the control, A, B and C groups. Note that DDO1002-treatment have no significant changes on kidney morphology, ($n \geq 3$).

(E) Serum levels of serum creatinine (CR) in control and DDO1002-treated mice.

(F) The percentage of body weight was recorded and calculated (initial body weight was 100%).

(G) The number of red blood cells (RBCs), haemoglobin (HGB), and monocytes (MON) in peripheral blood (*$P < 0.05$, **$P < 0.01$ vs. control). Values represent the mean ± SEM of the numbers of peripheral blood cells.

(H) Relative mRNA levels of *Ho-1*, *Nqo-1*, and *Gclm* in BM cells from control and DDO1002-treated aging mice (*$P < 0.05$, ****$P < 0.0001$ vs. control).

(I) Western blot analysis of Ho-1, Nqo-1, and Gclm in BM cells from control and DDO1002-treated aging mice.

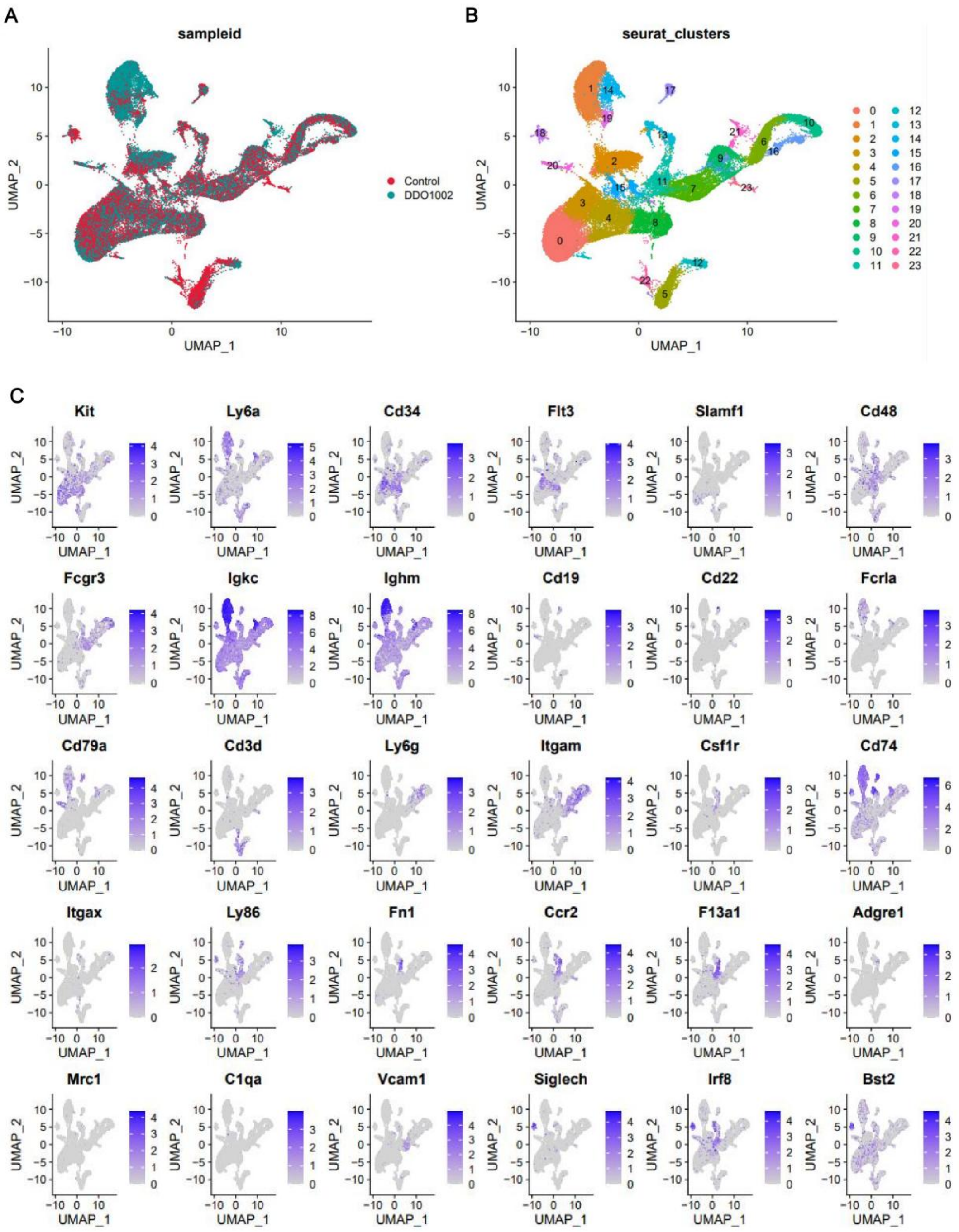

Supplementary Figure 4. Characterisation of scRNA-seq cell clusters by expression of marker genes.

(A) UMAP plot showing that no significant batch effects were observed between DDO1002-treated and control samples.

(B) UMAP plot showing unsupervised clusters.

(C) UMAP plot showing the expression of key marker genes in different clusters.

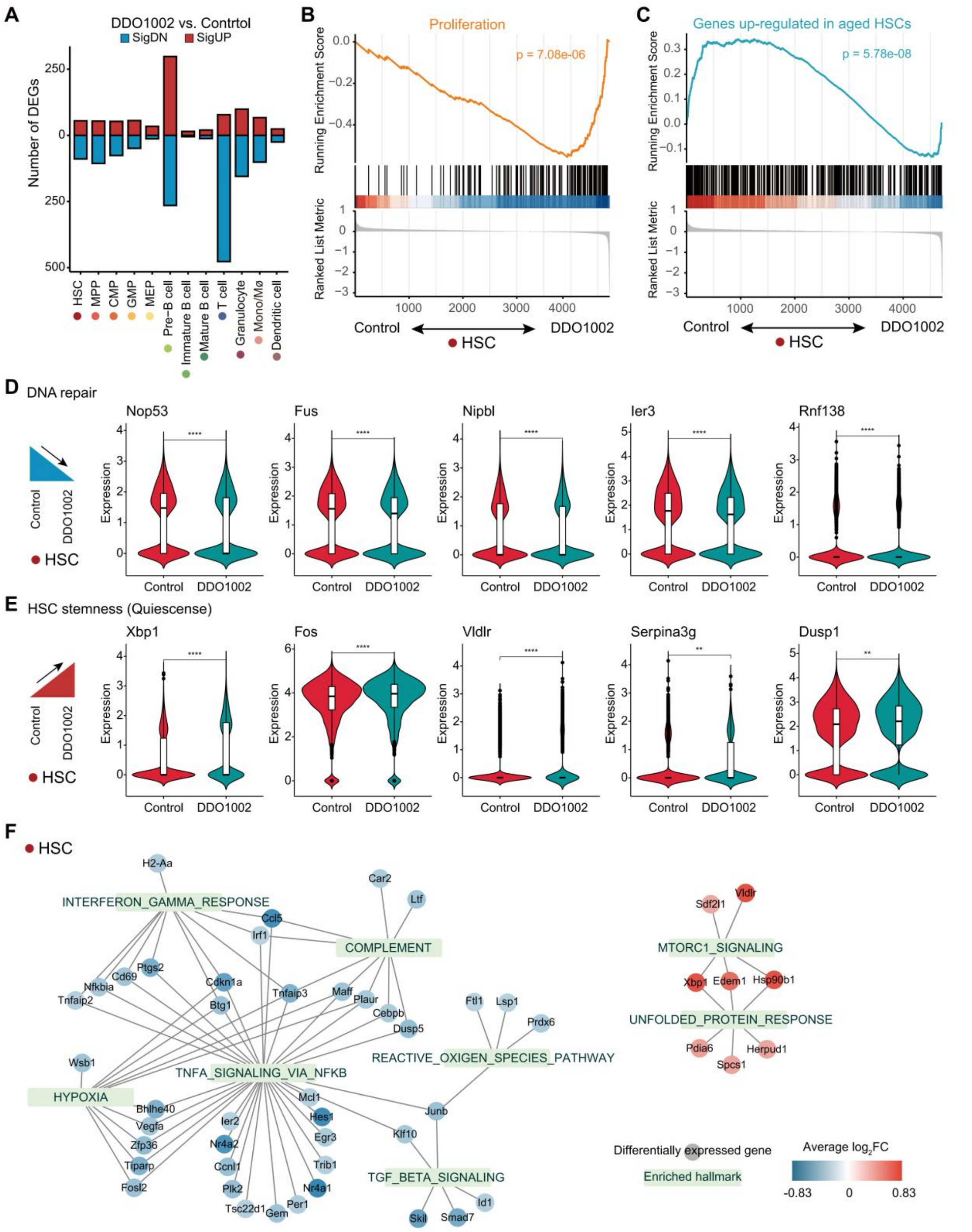

Supplementary Figure 5. DDO1002 treatment rescued age-related transcriptomic signature of aged HSCs.

(A) Bar graph showing the number of differentially expressed genes (DEGs) in each cell type after treatment with DDO1002.

(B) GSEA plot show upregulation of proliferation signature genes in

DDO1002-treated HSCs.

(C) GSEA plot show downregulation of genes up-regulated in aged HSCs (GSE47819) in DDO1002-treated HSCs.

(D) Violin plots show downregulation of DNA repair-associated genes in DDO1002-treated HSCs.

(E) Violin plots show upregulation of HSC stemness-associated genes in DDO1002-treated HSCs.

(F) Network showing up- and down-regulated genes in DDO1002-treated HSCs and the hallmarks enriched by them. Circle, gene; rounded rectangle, enriched hallmark. Color of circle, average $\log_2$(fold-change) of genes.

Table S1 Primers of Real-time quantitative PCR

	primer (5’-3’)
h-HO-1-F	ATGGCCTCCCTGTACCACATC
h-HO-1-R	TGTTGCGCTCAATCTCCTCCT
h-NQO1-F	CGCAGACCTTGTGATATTCCAG
h-NQO1-R	CGTTTCTTCCATCCTTCCAGG
h-GCLM-F	TTGGAGTTGCACAGCTGGATTC
h-GCLM-R	TGGTTTTACCTGTGCCCACTG
h-GAPDH-F	TCAACGACCACTTTGTCAAGCTCA
h-GAPDH-R	GCTGGTGGTCCAGGGGTCTTACT
m-Ho-1-F	GCTGGTGATGGCTTCCTTGTA
m-Ho-1-R	ACCTCGTGGAGACGCTTTACAT
m-Nqo1-F	ACGACAACGGTCCTTTCCAGA
m-Nqo1-R	CAGAAACGCAGGATGCCACT
m-Gclm-F	AGGAGCTTCGGGACTGTATCC
m-Gclm-R	GGAAACTCCCTGACTAAATCGG
m-β-actin-F	ATCTGGCACCACACCTTC
m-β-actin-R	AGCCAGGTCCAGACGCA
